# Supplementary material for: Catecholaminergic challenge uncovers distinct Pavlovian and instrumental mechanisms of motivated (in)action
Source: eLife. 2017 May 15;6:e22169. doi: 10.7554/eLife.22169 (PMC5432212; doi:10.7554/eLife.22169)
Supplement: Source code 1. — This zip-file contains source code for (1) deblinding, (2) descriptives & demographics, (3) mood ratings, (4) motivational Go-NoGo task, and a README.txt file with description of the source code and code structure. DOI: http://dx.doi.org/10.7554/eLife.22169.019 [file elife-22169-code1.zip › 1. deblinding/AKF2049 randomisatielijst_DEBLIND.pdf]

Randnr	Periode 1	Periode 2
1	1	0
2	1	0
3	0	1
4	0	1
5	1	0
6	1	0
7	0	1
8	0	1
9	1	0
10	0	1
11	0	1
12	1	0
13	1	0
14	0	1
15	1	0
16	0	1
17	1	0
18	0	1
19	0	1
20	1	0
21	1	0
22	1	0
23	0	1
24	0	1
25	0	1
26	1	0
27	0	1
28	1	0
29	1	0
30	0	1
31	1	0
32	0	1
33	0	1
34	0	1
35	1	0
36	1	0
37	1	0
38	0	1
39	0	1
40	1	0
41	1	0
42	1	0
43	0	1
44	0	1
45	1	0
46	0	1
47	1	0
48	0	1

0 = placebo
1 = actief (methylfenidaat 20mg)

Drs M Welzen [signature] 21 MAART 2014 21-3-2014 → check. SV

49	0	1
50	0	1
51	1	0
52	1	0
53	1	0
54	1	0
55	0	1
56	0	1
57	1	0
58	0	1
59	0	1
60	1	0
61	1	0
62	0	1
63	1	0
64	0	1
65	1	0
66	0	1
67	0	1
68	1	0
69	1	0
70	0	1
71	0	1
72	1	0
73	1	0
74	1	0
75	0	1
76	0	1
77	1	0
78	0	1
79	1	0
80	0	1
81	1	0
82	0	1
83	0	1
84	1	0
85	1	0
86	1	0
87	0	1
88	0	1
89	0	1
90	1	0
91	1	0
92	0	1
93	0	1
94	1	0
95	1	0
96	0	1
97	0	1

Drs M Welzen 1 MAART 2014 21-3-2014 → check SV

98	1	0
99	1	0
100	0	1
101	0	1
102	0	1
103	1	0
104	0	1
105	1	0

Drs M Welzen 2 1 MAART 2014 21-3-2014 → check SV
